# Eczema-like Granular Parakeratosis Exhibits Inflammatory Keratinocyte Remodeling and Aberrant Epidermal Differentiation

**DOI:** 10.3390/ijms27188418

**Published:** 2026-09-21

**Authors:** Shihui Zhou, Yijun Yang, Jingjun Zhao, Yifeng Guo

**Affiliations:** Dermatology Center, Xinhua Hospital, Shanghai Jiao Tong University School of Medicine, Shanghai 200092, China

**Keywords:** dermatitis, granular parakeratosis, keratinocytes, single-cell analysis, benzalkonium compounds

## Abstract

Granular parakeratosis (GP) is an uncommon keratinization disorder that predominantly affects intertriginous areas and may clinically mimic atopic dermatitis or psoriasis. Histologically, GP is characterized by compact hyperkeratosis, parakeratosis, and retention of keratohyalin granules within the stratum corneum, although its histopathological spectrum is heterogeneous. Previous studies have consisted mainly of case reports, while mechanistic investigations remain limited, and whether GP represents a purely cornification-related abnormality is unclear. During and after the coronavirus disease 2019 (COVID-19) pandemic, increased use of benzalkonium chloride (BAC)-containing laundry disinfectants coincided with more patients presenting with recurrent erythema and scaling that responded poorly to conventional treatment, prompting renewed attention to this disease. We performed single-cell RNA sequencing of lesional skin from three patients with GP and matched skin samples from three healthy controls, followed by keratinocyte subclustering, pseudotime analysis, and cell–cell communication analysis. Key findings were further validated by immunofluorescence in an independent cohort of four patients with GP and three healthy controls. HaCaT cells were exposed to BAC and analyzed by the Cell Counting Kit-8 (CCK-8) assay and bulk RNA sequencing. Single-cell analysis revealed the expansion of inflammatory keratinocytes in GP lesions. Pathway enrichment showed the activation of innate immune and antimicrobial responses and dysregulation of keratinocyte differentiation. *S100A8/S100A9* were increased in GP keratinocytes and validated at the protein level. BAC induced interleukin-1 (IL-1)-related inflammatory activation and cellular stress responses, suggesting that GP is an exposure-associated inflammatory epidermal differentiation disorder.

## 1. Introduction

Granular parakeratosis (GP), also referred to as hyperkeratotic flexural erythema, is an acquired disorder of epidermal keratinization that predominantly affects intertriginous and flexural sites, including the axillae, groin, and popliteal fossae [1]. Clinically, GP typically presents as erythematous to brownish hyperkeratotic papules or plaques, often accompanied by scaling, pruritus, burning, or pain [2]. Because these manifestations overlap substantially with atopic dermatitis, fungal infection, inverse psoriasis, and intertrigo, GP is frequently underrecognized and may be repeatedly misdiagnosed before histopathological confirmation.

The diagnostic hallmark of GP is compact hyperkeratosis with parakeratosis and the retention of keratohyalin granules within the stratum corneum [3]. Although this histological pattern supports a disorder of cornification, GP exhibits clinicopathological heterogeneity that is not fully explained by abnormal keratinization alone. In addition to the characteristic stratum corneum changes, epidermal hyperplasia, interface alteration, and superficial perivascular inflammatory infiltrates have been described in a subset of lesions [4]. These observations raise the possibility that inflammatory epidermal remodeling may contribute to the development or maintenance of GP.

The etiology of GP remains incompletely understood. Reported triggers include deodorants, antiperspirants, occlusion, friction, sweating, and topical or laundry products containing benzalkonium chloride (BAC), a quaternary ammonium compound widely used in disinfectants and cleansing products. Among these, BAC has emerged as one of the most frequently implicated and clinically relevant triggers of GP [5,6,7]. Although GP has traditionally been considered rare, with an estimated incidence of approximately 0.005% [8], increased use of disinfectant-containing products during and after the coronavirus disease 2019 (COVID-19) pandemic may have increased clinical encounters with GP-like eruptions [9]. This changing exposure landscape highlights the need to clarify how environmental triggers may contribute to abnormal epidermal differentiation.

Despite increasing clinical recognition, the molecular basis of GP remains largely undefined. Existing studies have mainly established its clinical morphology, histopathological spectrum, and potential external triggers, whereas the cellular and molecular mechanisms underlying GP remain poorly investigated [10,11]. In particular, it remains unclear whether BAC-associated GP should be understood simply as a keratinization disorder triggered by external products, or as an exposure-associated inflammatory epidermal differentiation abnormality.

In this study, we combined single-cell transcriptomic profiling, pseudotime trajectory analysis, cell–cell communication analysis, tissue immunofluorescence validation, and an in vitro BAC-exposed keratinocyte model. We aimed to define the cellular and molecular features of GP lesions and to explore whether BAC exposure can induce keratinocyte inflammatory and stress-response programs that may contribute to aberrant epidermal differentiation.

## 2. Results

### 2.1. Single-Cell Transcriptomic Profiling Reveals Altered Cellular Composition in Granular Parakeratosis Lesions

**Figure 1:**
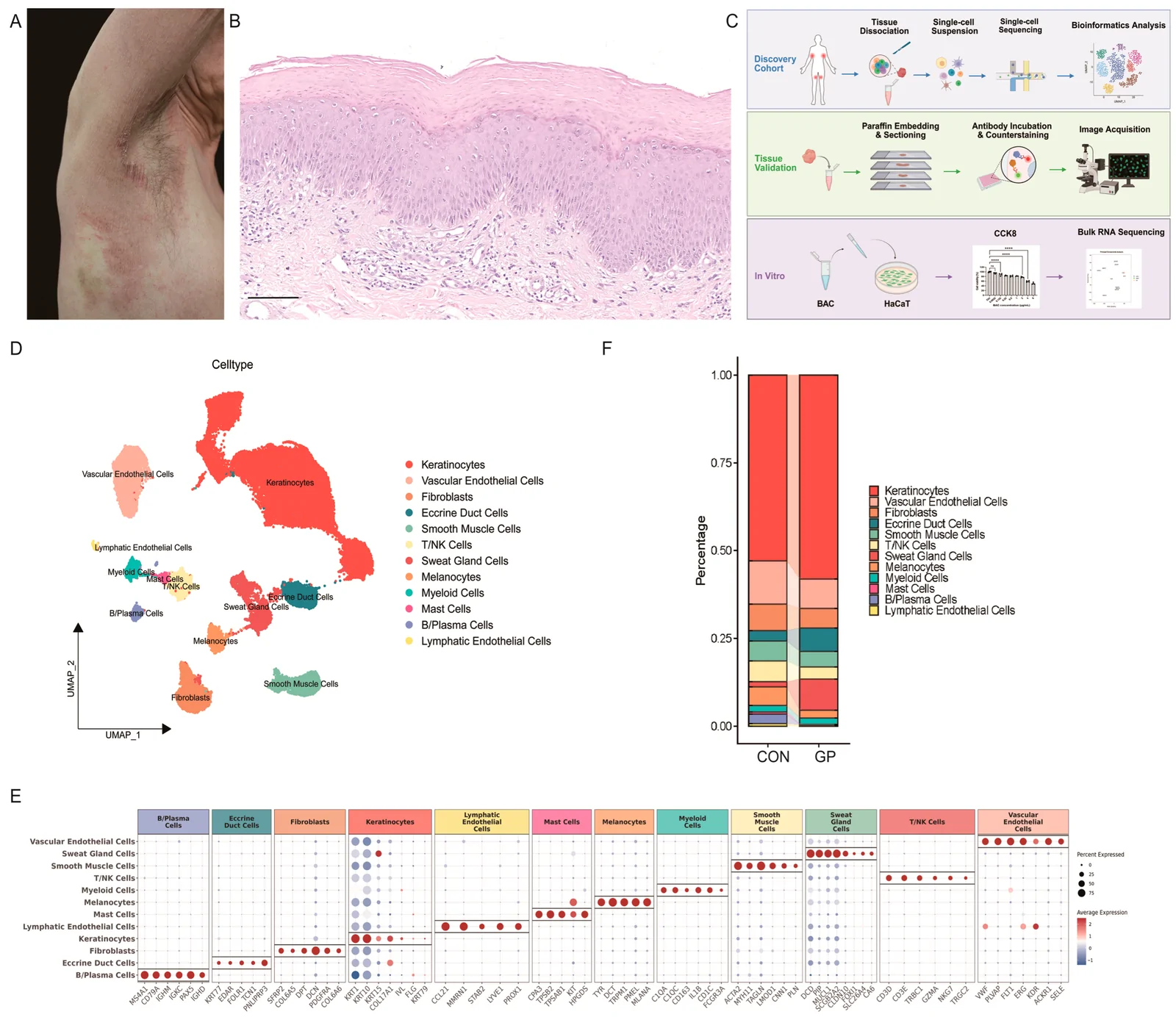
Clinical presentation, study design, and single-cell transcriptomic landscape of granular parakeratosis. (**A**) Representative clinical image showing erythematous plaques with scaling in the axillary region. (**B**) Representative hematoxylin and eosin (H&E)-stained section of GP lesional skin showing hyperkeratosis with parakeratosis, mild epidermal hyperplasia, superficial dermal inflammatory infiltrates, and retention of keratohyalin granules within the stratum corneum (scale bar = 200 μm). (**C**) Schematic overview of the study design, including single-cell RNA sequencing of GP lesional skin and healthy control skin, immunofluorescence validation, and in vitro benzalkonium chloride exposure experiments. ns, not significant; **** *p* < 0.0001. Created with BioRender.com. (**D**) UMAP visualization of the 12 major skin cell populations identified by single-cell RNA sequencing. (**E**) Dot plot showing representative canonical marker genes used for cell-type annotation. (**F**) Relative cellular composition of healthy control and GP lesional skin.

GP typically presents as recurrent erythematous, scaly plaques in intertriginous areas, including the axillae and groin. A representative patient showed erythematous plaques with scaling in the axillary region (Figure 1A). Representative hematoxylin and eosin (H&E) staining of GP lesional skin showed hyperkeratosis with parakeratosis and retention of keratohyalin granules within the stratum corneum. Mild epidermal hyperplasia and superficial dermal inflammatory infiltrates were also observed (Figure 1B). To investigate the cellular basis of GP, we performed single-cell RNA sequencing (scRNA-Seq) of lesional GP skin and skin from healthy controls (CON), followed by cell-type annotation, keratinocyte subclustering, trajectory analysis, cell–cell communication analysis, tissue-level validation, and in vitro BAC exposure experiments (Figure 1C).

After quality control and unsupervised clustering, a total of 72,676 cells were retained for subsequent analysis, with an average of 2025 genes and 5703 transcripts detected per cell. Using Uniform Manifold Approximation and Projection (UMAP)-based dimensionality reduction, we identified 12 major skin cell populations, including keratinocytes, T/NK cells, B/plasma cells, myeloid cells, vascular endothelial cells, lymphatic endothelial cells, eccrine duct cells, fibroblasts, smooth muscle cells, melanocytes, mast cells, and sweat gland cells (Figure 1D). Cell identities were assigned according to canonical marker genes. Keratinocytes expressed *KRT1*, *KRT10*, *KRT15* and *COL17A1*; T/NK cells expressed *CD3D*, *CD3E*, and *TRBC1*; B/plasma cells expressed *CD79A* and *MS4A1*; and myeloid cells expressed *C1QA*, *C1QC* and *CD1C.* Endothelial cells, fibroblasts, melanocytes, mast cells, and sweat gland cells were similarly annotated using established lineage markers (Figure 1E).

Comparison of cellular composition between healthy control skin and GP lesional skin revealed an apparent redistribution of major skin cell populations (Figure 1F). Keratinocytes constituted the dominant epidermal population in both groups, supporting a keratinocyte-centered analysis of GP pathogenesis. Given the defining histopathological abnormalities in epidermal cornification and the observed remodeling of the lesional cellular landscape, we next focused on keratinocyte states and their relationship with the microenvironment.

### 2.2. Keratinocyte Subclustering Identifies Expansion of Inflammatory Keratinocytes in GP Lesions

**Figure 2:**
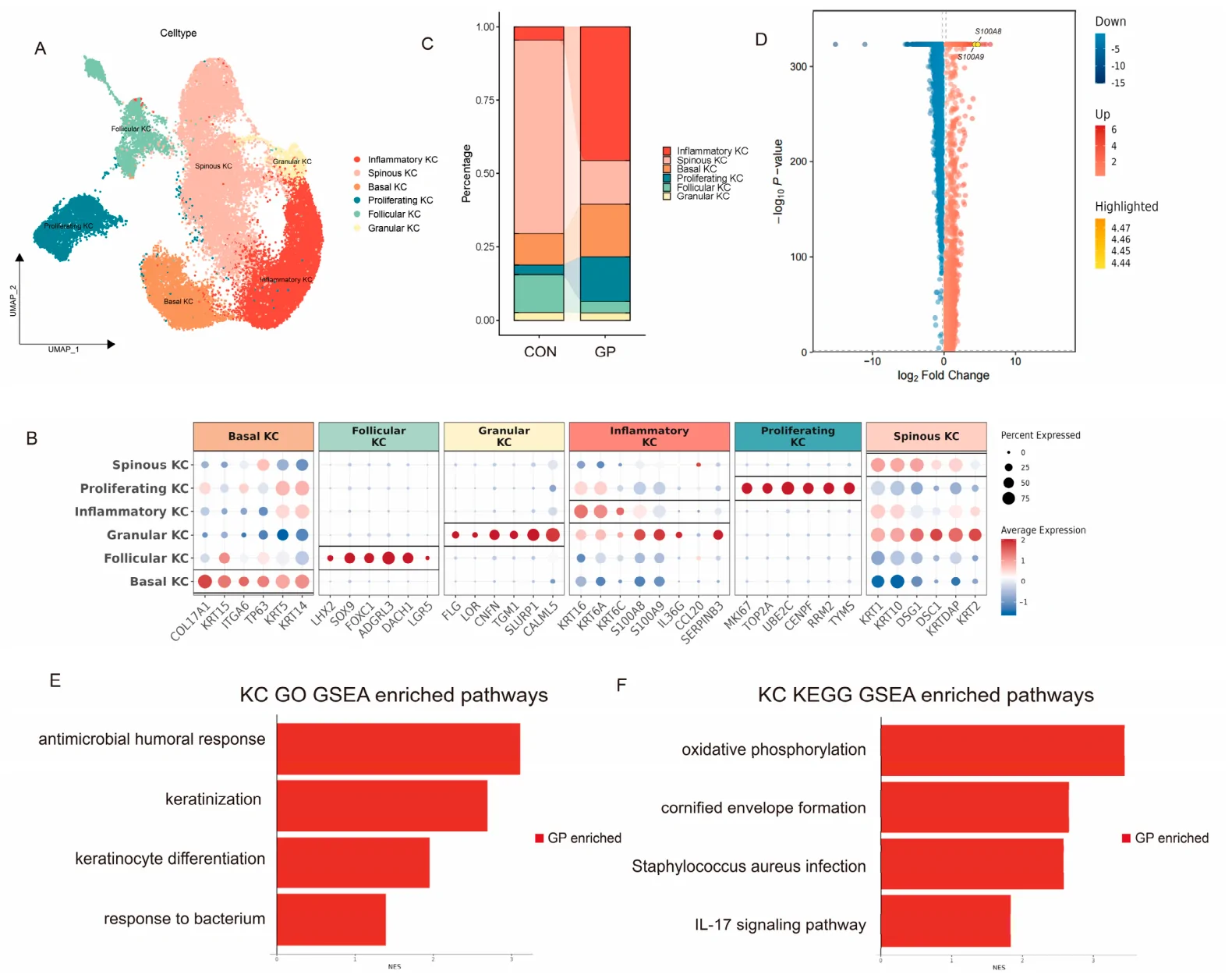
Keratinocyte subclustering reveals inflammatory keratinocyte remodeling in granular parakeratosis. (**A**) UMAP visualization of keratinocyte subclusters identified after reclustering of the keratinocyte compartment. (**B**) Dot plot showing representative marker genes across keratinocyte subpopulations. (**C**) Relative proportions of keratinocyte subpopulations in healthy control skin and GP lesional skin. (**D**) Volcano plot showing differentially expressed genes in GP keratinocytes compared with control keratinocytes. (**E**) GO enrichment analysis of GP keratinocytes showing enrichment of antimicrobial humoral response, keratinization, keratinocyte differentiation, and response to bacterium. (**F**) KEGG enrichment analysis showing enrichment of oxidative phosphorylation, cornified envelope formation, IL-17 signaling, and infection-related pathways in GP keratinocytes.

Given that GP is histologically defined by abnormal epidermal cornification, we next focused on the keratinocyte compartment. Reclustering of keratinocytes identified multiple differentiation-stage and state-specific keratinocyte populations, including basal keratinocytes, proliferating keratinocytes, spinous keratinocytes, granular keratinocytes, follicular keratinocytes, and inflammatory keratinocytes (Figure 2A). The marker gene dot plot further confirmed the transcriptional identity of each keratinocyte subpopulation (Figure 2B). Basal keratinocytes were characterized by the expression of *COL17A1*, *KRT15* and *ITGA6*. Granular keratinocytes showed enrichment of terminal differentiation- and cornification-associated genes, such as *SLURP1*, *CALML5*, *CNFN*, and *TGM1*. Proliferating keratinocytes were marked by cell-cycle genes, including *UBE2C*, *MKI67*, *CENPF*, and *TOP2A*. Inflammatory keratinocytes showed prominent expression of inflammation- and stress-associated epithelial genes, including *KRT16*, *S100A8*, and *S100A9*. These marker patterns supported reliable annotation of distinct keratinocyte states and highlighted the emergence of an inflammatory keratinocyte population in GP lesions.

Comparison of keratinocyte composition between the control and GP skin showed a striking shift in keratinocyte states. In control skin, spinous keratinocytes constituted the dominant keratinocyte population, consistent with a relatively preserved epidermal differentiation program. In contrast, GP lesions showed a marked enrichment of inflammatory keratinocytes, accompanied by a relative reduction in normally differentiated keratinocyte subpopulations (Figure 2C). These findings suggest that GP is associated with keratinocyte state remodeling rather than an isolated abnormality of the stratum corneum.

Differential expression analysis between GP and healthy control keratinocytes further supported this inflammatory remodeling. Volcano plot analysis demonstrated marked upregulation of *S100A8* and *S100A9* in GP keratinocytes, together with other genes associated with keratinocyte activation and inflammatory epidermal responses (Figure 2D). To investigate the biological programs underlying these transcriptional changes, we performed pathway enrichment analysis. Gene Ontology (GO) analysis revealed significant enrichment of antimicrobial humoral response, keratinization, keratinocyte differentiation, and response to bacterium in GP keratinocytes (Figure 2E). Kyoto Encyclopedia of Genes and Genomes (KEGG) pathway analysis further showed enrichment of cornified envelope formation, interleukin-17 (IL-17) signaling pathway, oxidative phosphorylation, and Staphylococcus aureus infection-related signatures (Figure 2F). Together, these data indicate that GP keratinocytes exhibit a combined inflammatory and abnormal differentiation program, supporting the concept that GP is not merely a disorder of cornification, but involves inflammation-associated keratinocyte remodeling.

### 2.3. S100A8/S100A9-Positive Inflammatory Keratinocytes Are Enriched in GP Lesions and Validated at the Tissue Level

**Figure 3:**
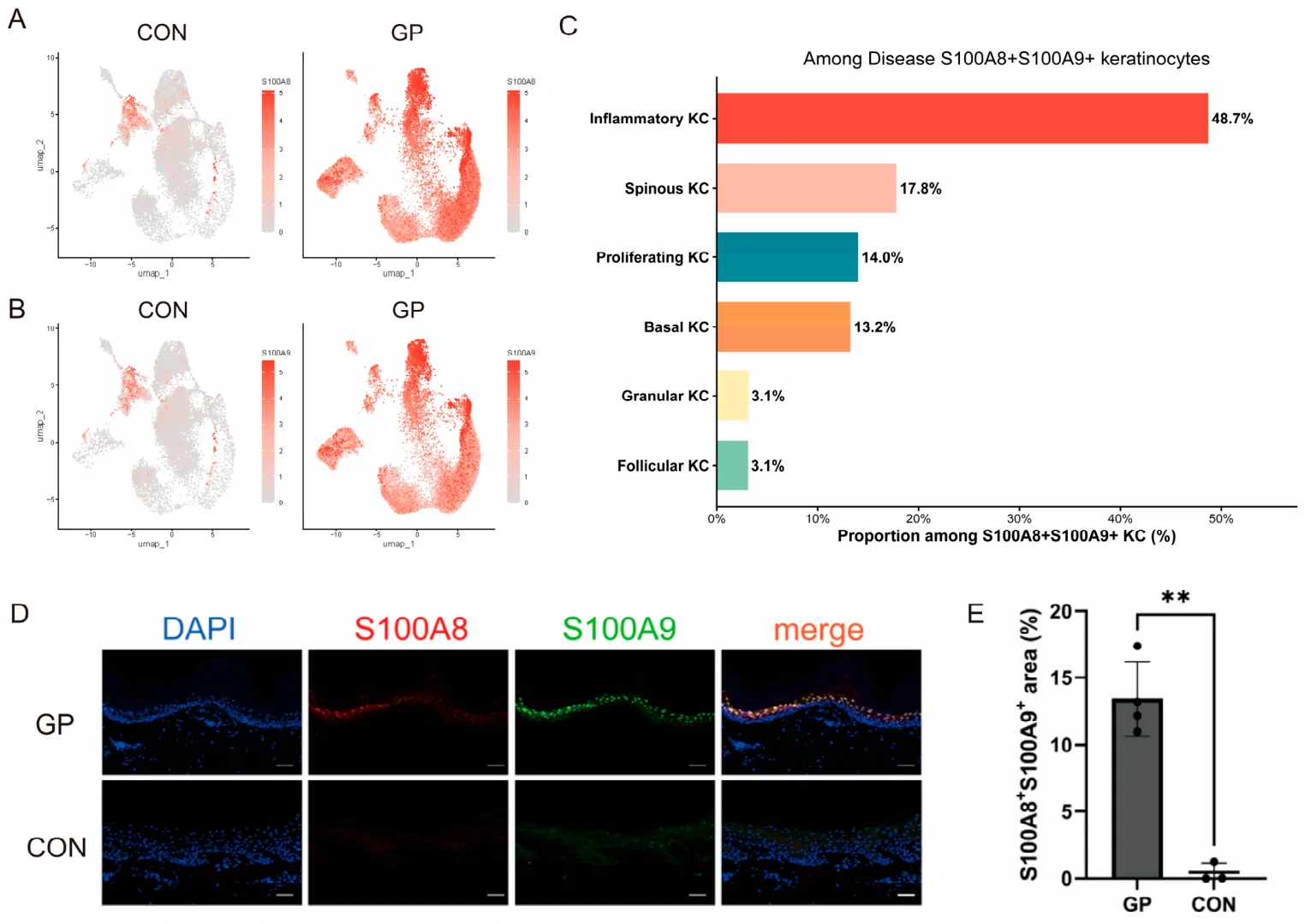
S100A8/S100A9-positive inflammatory keratinocytes are enriched in granular parakeratosis lesions and validated by immunofluorescence. (**A**) Feature plots showing *S100A8* expression in keratinocytes from healthy control skin and GP lesional skin. (**B**) Feature plots showing *S100A9* expression in keratinocytes from healthy control skin and GP lesional skin. (**C**) Distribution of *S100A8/S100A9* double-positive keratinocytes across keratinocyte subclusters in GP lesions. (**D**) Representative images of immunofluorescence staining of S100A8/S100A9 double-positive keratinocytes in GP and healthy controls as identified by S100A8 (red) and S100A9 (green). Nuclei were counterstained with 4′,6-diamidino-2-phenylindole (DAPI; blue). Scale bar = 50 μm. (**E**) Quantification of S100A8/S100A9 double-positive keratinocyte area percentage in GP patients and healthy controls. Data are presented as mean ± standard deviation (SD). Statistical significance was determined by an unpaired *t*-test. **, *p* < 0.01.

Based on the differential expression analysis, we next examined the spatial distribution and keratinocyte-state origin of *S100A8* and *S100A9* expression. Feature plots demonstrated minimal *S100A8* and *S100A9* expression in the control keratinocytes, whereas both genes were highly expressed in GP keratinocytes (Figure 3A,B).

To further define the cellular source of this signal, we analyzed the composition of *S100A8/S100A9* double-positive keratinocytes in GP lesions. Among the double-positive keratinocytes, the largest fraction was assigned to inflammatory keratinocytes, accounting for 48.7% of this population, followed by spinous keratinocytes and proliferating keratinocytes (Figure 3C). These findings indicate that *S100A8/S100A9* expression in GP lesions primarily reflects an inflammatory keratinocyte state, rather than a nonspecific increase across the entire epidermal compartment.

We next validated this single-cell finding at the tissue level using immunofluorescence staining in GP lesions and healthy control skin. Compared with the healthy controls, the GP epidermis showed markedly stronger S100A8 and S100A9 signals (Figure 3D). Quantitative analysis confirmed a significantly increased S100A8/S100A9 double-positive epidermal area in GP lesions compared with the controls (Figure 3E). Together, these data demonstrate that GP lesions are characterized by the expansion of S100A8/S100A9-positive inflammatory keratinocytes, providing tissue-level validation of the inflammatory keratinocyte remodeling identified by single-cell transcriptomics.

### 2.4. Pseudotime Analysis Reveals Altered Keratinocyte Differentiation Trajectories in GP Lesions

**Figure 4:**
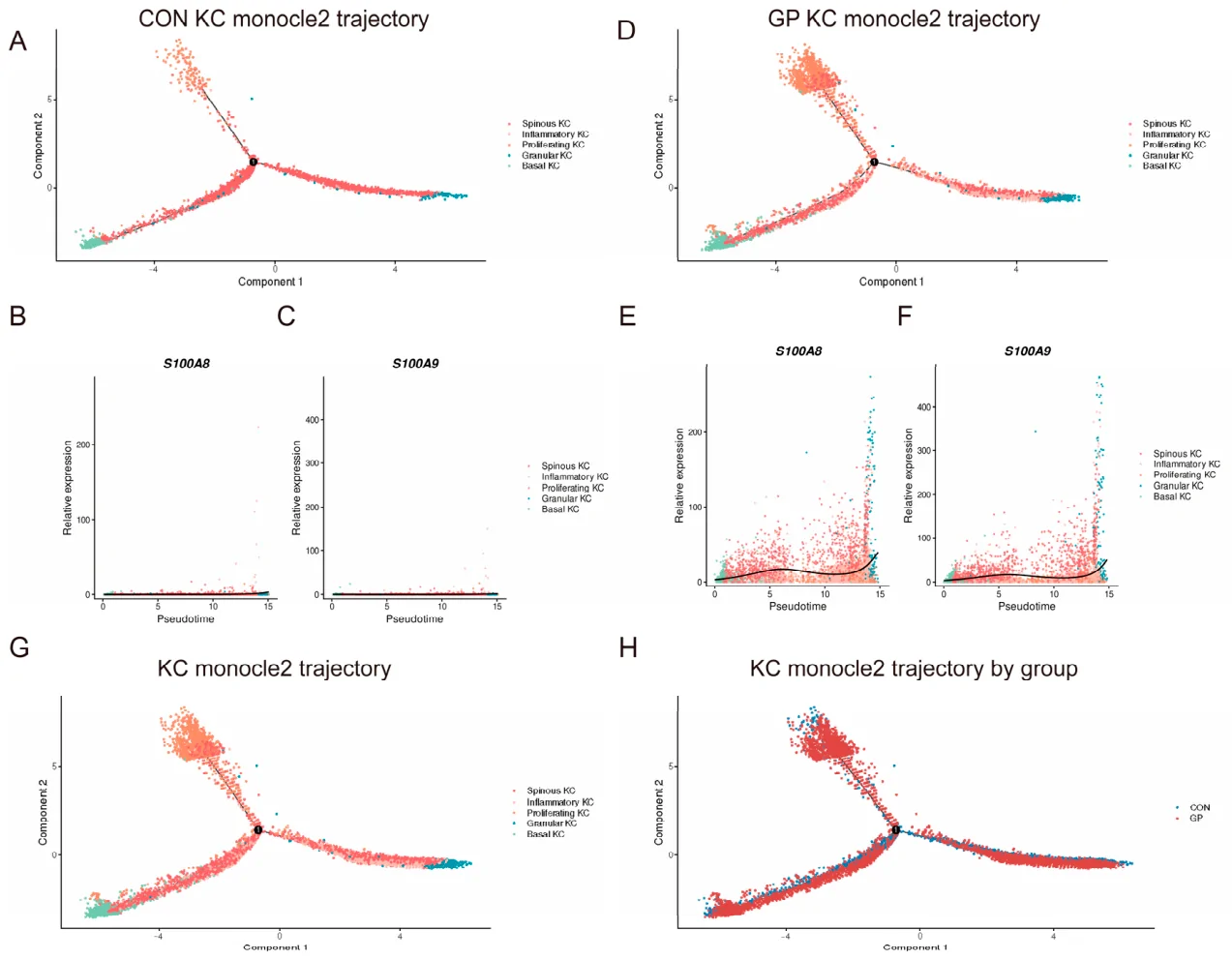
Pseudotime analysis reveals altered keratinocyte differentiation trajectories in granular parakeratosis. (**A**) Pseudotime trajectory of keratinocytes from healthy control skin, showing a relatively continuous differentiation path from basal keratinocytes toward spinous and granular keratinocyte states. The black line indicates the inferred pseudotime trajectory. (**B**,**C**) Expression dynamics of *S100A8* and *S100A9* along pseudotime in control keratinocytes. (**D**) Pseudotime trajectory of keratinocytes from GP lesional skin, showing accumulation of inflammatory keratinocytes along a trajectory segment spanning spinous and granular keratinocyte states. (**E**,**F**) Expression dynamics of *S100A8* and *S100A9* along pseudotime in GP keratinocytes. (**G**) Combined pseudotime trajectory of control and GP keratinocytes colored by keratinocyte subtype. (**H**) Combined pseudotime trajectory colored by sample group.

To determine whether the inflammatory keratinocyte state in GP was associated with altered epidermal differentiation, we performed pseudotime trajectory analysis of the keratinocytes. In healthy control skin, keratinocytes were distributed along an interconnected inferred trajectory, with basal, spinous, and granular keratinocytes occupying different trajectory regions (Figure 4A). Along this trajectory, *S100A8* and *S100A9* expression remained low overall, consistent with a predominantly homeostatic differentiation program (Figure 4B,C).

In GP lesions, inflammatory keratinocytes were concentrated along the trajectory segment connecting spinous and granular keratinocyte states, suggesting an association between inflammatory activation and the terminal differentiation program (Figure 4D). *S100A8* and *S100A9* expression was substantially stronger in GP keratinocytes than in the control keratinocytes (Figure 4E,F).

We further examined the control and GP keratinocytes within a combined pseudotime framework. Visualization by keratinocyte subtype and sample group revealed overlapping distributions of the control and GP keratinocytes across the inferred trajectory (Figure 4G,H). Together, the pseudotime analysis supports that inflammatory activation accompanies differentiation-associated keratinocyte states in GP lesions.

### 2.5. Cell-Cell Communication Analysis Suggests Keratinocyte-Centered Inflammatory and Repair Signaling in GP Lesions

**Figure 5:**
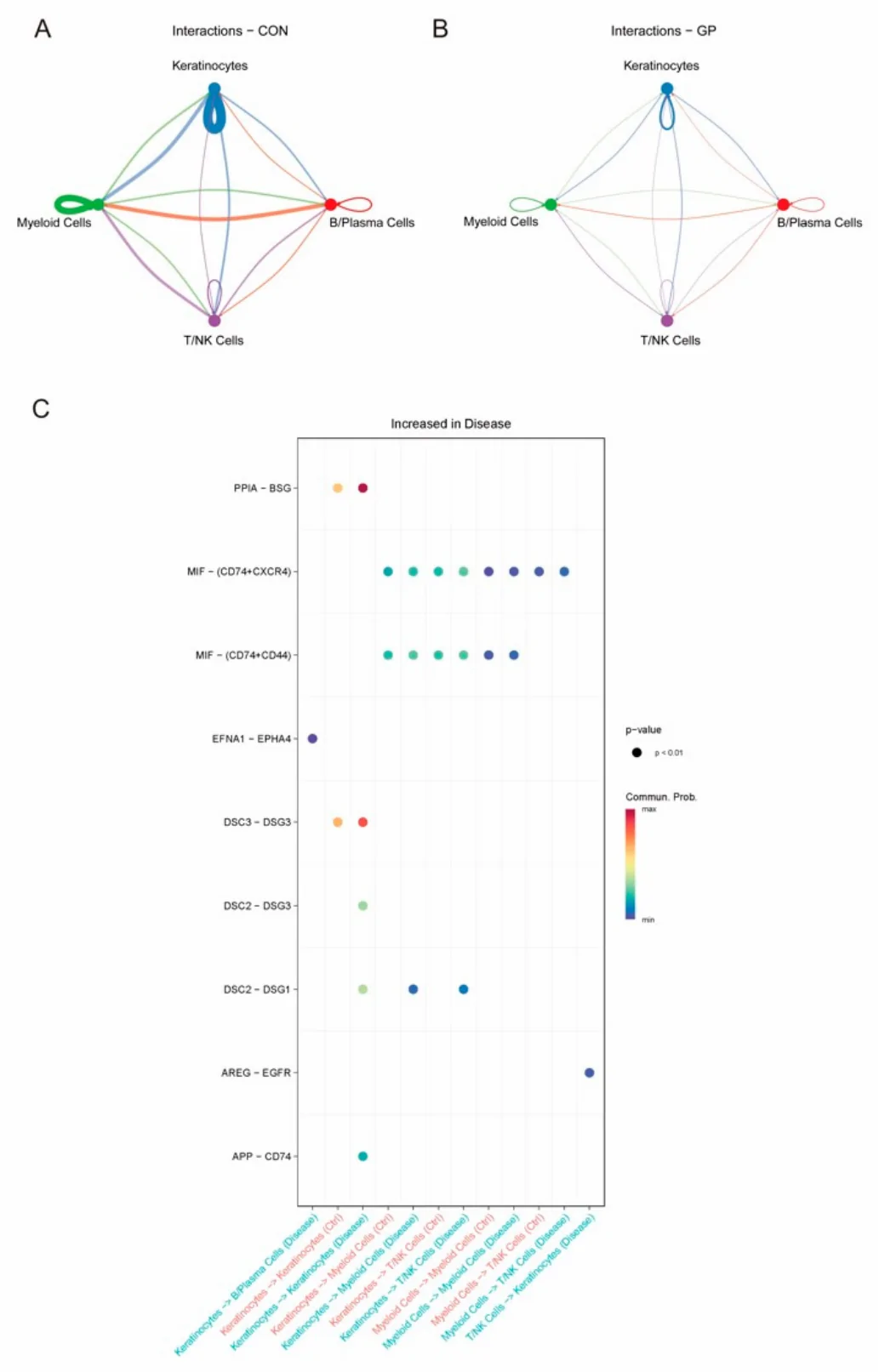
Cell-cell communication analysis reveals remodeling of keratinocyte-associated signaling networks in granular parakeratosis lesions. (**A**) Circle plots showing the predicted number of cell–cell communications among keratinocytes, T/NK cells, B/plasma cells, and myeloid cells in healthy controls. (**B**) Circle plots showing the predicted number of cell–cell communications among keratinocytes, T/NK cells, B/plasma cells, and myeloid cells in GP lesional skin. (**C**) Bubble plot showing selected altered keratinocyte-associated ligand–receptor interactions.

To investigate whether inflammatory keratinocyte remodeling in GP was accompanied by changes in intercellular signaling, we performed cell–cell communication analysis using CellChat. Comparison of GP and control skin revealed an overall reduction in the number of predicted interactions among keratinocytes, T/NK cells, B/plasma cells, and myeloid cells in GP lesions (Figure 5A,B). Despite this global reduction, keratinocytes represented a major communication hub in both conditions, consistent with their central role in epidermal homeostasis and lesion formation.

We next examined keratinocyte-associated ligand–receptor interactions that were selectively increased in GP skin. The most evident alterations involved keratinocyte-to-keratinocyte signaling, including increased PPIA–BSG and DSC3–DSG3 communication, together with GP-associated DSC2–DSG3, DSC2–DSG1, and APP–CD74 interactions. PPIA–BSG signaling has been implicated in inflammatory activation and keratinocyte proliferative responses [12], whereas desmocollin–desmoglein interactions mediate desmosomal adhesion and are closely associated with epidermal cohesion and keratinocyte differentiation [13,14]. AREG–EGFR signaling from T/NK cells to keratinocytes suggests a potential immune-to-epithelial proliferative and repair-associated signal [15], while EFNA1–EPHA4 signaling from keratinocytes to B/plasma cells indicates altered contact-dependent epithelial–immune communication (Figure 5C) [16]. Collectively, these selectively altered interactions suggest the remodeling of signaling processes associated with keratinocyte activation, intercellular adhesion, epidermal differentiation, and bidirectional epithelial–immune communication.

In addition to these selective alterations, macrophage migration inhibitory factor (MIF) signaling through the CD74–CXCR4 and CD74–CD44 receptor complexes was broadly detected across multiple communication routes involving keratinocytes, myeloid cells, and T/NK cells. The magnitude and direction of MIF-associated changes varied among sender–receiver cell pairs, suggesting cell-type-dependent redistribution rather than uniform activation of the MIF pathway.

Together with the keratinocyte subclustering and pseudotime findings, these results support coordinated remodeling of keratinocyte differentiation-associated states and intercellular communication in GP lesions.

### 2.6. Benzalkonium Chloride Induces Dose-Dependent Keratinocyte Injury and Inflammatory Stress-Response Programs In Vitro

**Figure 6:**
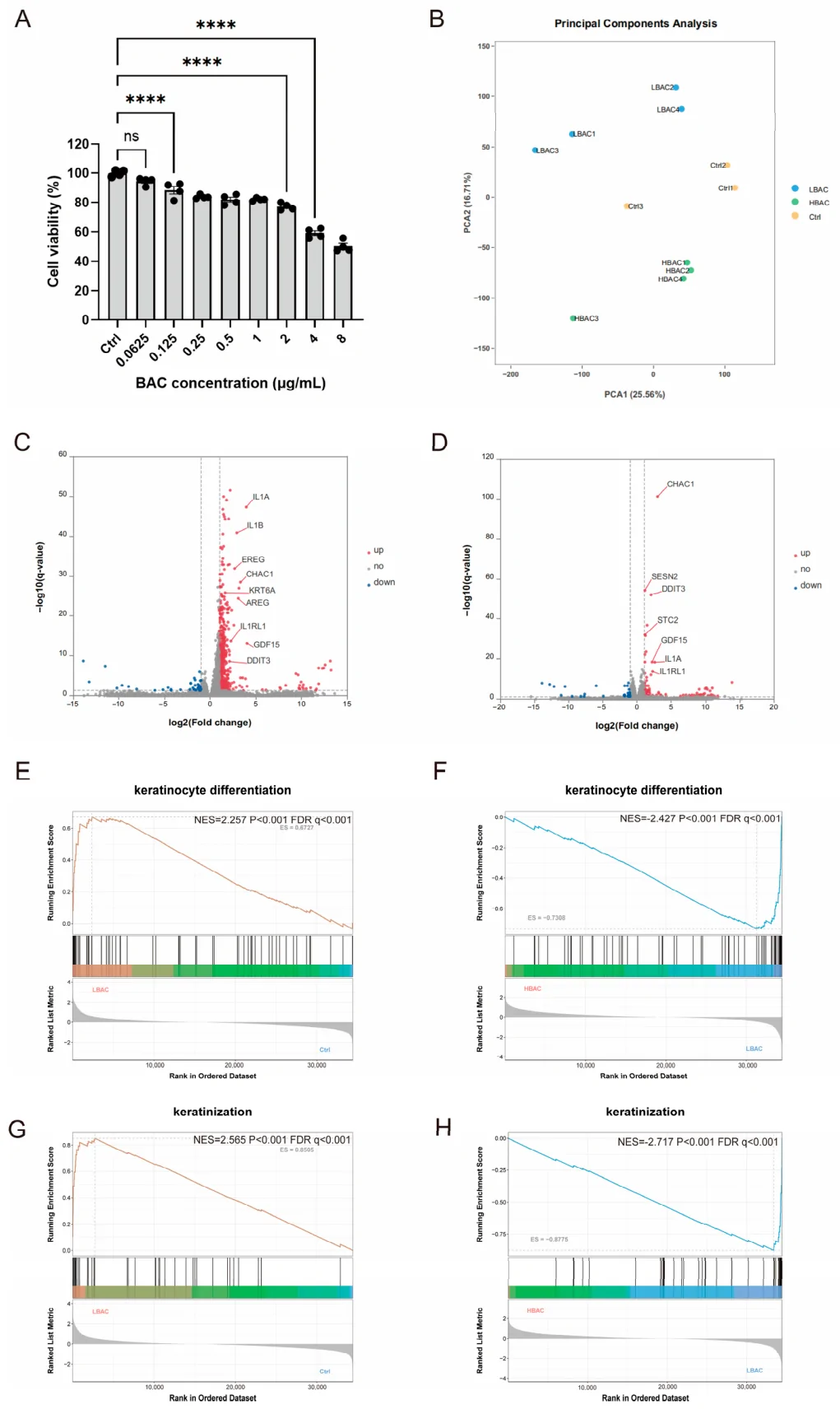
Benzalkonium chloride exposure induces inflammatory and stress-response programs and alters differentiation-associated transcriptional programs in HaCaT cells. (**A**) CCK-8 assay evaluating HaCaT cell viability following treatment with serial concentrations of benzalkonium chloride (0.0625, 0.125, 0.25, 0.5, 1, 2, 4, 8 μg/mL). Data are presented as mean ± SD; ns, not significant; **** *p* < 0.0001. (**B**) Principal component analysis of bulk RNA sequencing profiles from control, low-concentration BAC, and high-concentration BAC groups. (**C**) Volcano plot showing differentially expressed genes in low-concentration BAC-treated HaCaT cells compared with controls. Red, gray, and blue dots indicate upregulated, non-significant, and downregulated genes, re-spectively. The dashed lines indicate the DEG thresholds (|log2FC| = 1 and FDR = 0.05). (**D**) Volcano plot showing differentially expressed genes in high-concentration BAC-treated HaCaT cells compared with controls. (**E**) GSEA of the keratinocyte differentiation gene set in LBAC-treated cells compared with controls. (**F**) GSEA of the keratinocyte differentiation gene set in HBAC-treated cells compared with LBAC-treated cells. (**G**) GSEA of the keratinization gene set in LBAC-treated cells compared with controls. (**H**) GSEA of the keratinization gene set in HBAC-treated cells compared with LBAC-treated cells. LBAC, low-concentration BAC. HBAC, high-concentration BAC.

Given the clinical association between GP and exposure to BAC-containing products, we next investigated whether BAC could induce keratinocyte injury and inflammatory stress responses in vitro. HaCaT keratinocytes were treated with different concentrations of BAC, and cell viability was assessed using the Cell Counting Kit-8 (CCK-8) assay. BAC reduced HaCaT cell viability in a dose-dependent manner, with a significant decrease observed from 0.125 μg/mL (Figure 6A). Based on the viability curve, low-concentration BAC (LBAC, 2 μg/mL) and high-concentration BAC (HBAC, 4 μg/mL) exposure conditions were selected for subsequent bulk RNA sequencing.

Principal component analysis of the transcriptomic profiles showed separation among the control, low-concentration BAC, and high-concentration BAC groups, indicating that BAC exposure induced dose-associated transcriptional changes in HaCaT cells (Figure 6B). In the low-concentration BAC group, volcano plot analysis revealed the upregulation of genes related to interleukin-1 (IL-1)-associated inflammation, keratinocyte activation, and early stress responses, including *IL1A*, *IL1B*, *IL1RL1*, *AREG*, *EREG*, *KRT6A*, *CHAC1*, *DDIT3*, and *GDF15* (Figure 6C). In the high-concentration BAC group, the transcriptional response shifted toward a stronger cellular stress and damage-adaptation program, characterized by prominent upregulation of *CHAC1*, *DDIT3*, *SESN2*, *STC2*, *GDF15*, *IL1A*, and *IL1RL1* (Figure 6D).

To further determine whether BAC exposure affected keratinocyte differentiation-associated programs, we performed gene set enrichment analysis (GSEA) focusing on keratinocyte differentiation and keratinization. Compared with the control cells, LBAC-treated cells showed significant positive enrichment of the keratinocyte differentiation gene set (normalized enrichment score (NES) = 2.257, false discovery rate (FDR) q < 0.001) and the keratinization gene set (NES = 2.565, FDR q < 0.001) (Figure 6E,G). In contrast, both gene sets were negatively enriched in HBAC-treated cells compared with LBAC-treated cells, with NES values of −2.427 for keratinocyte differentiation and −2.717 for keratinization (FDR q < 0.001 for both) (Figure 6F,H). The reversal in enrichment direction between these comparisons indicates concentration-dependent remodeling of differentiation- and keratinization-associated transcriptional programs.

Taken together, these findings demonstrate that BAC exposure induces concentration-dependent transcriptional remodeling in HaCaT keratinocytes. LBAC exposure was characterized by IL-1-associated inflammatory activation and the enrichment of keratinocyte differentiation- and keratinization-related gene sets, whereas HBAC exposure elicited a more pronounced cellular stress and injury-adaptation response, accompanied by negative enrichment of these differentiation-associated programs relative to LBAC. Thus, BAC exposure not only activates inflammatory and stress responses, but also perturbs transcriptional programs involved in keratinocyte differentiation and keratinization, providing a molecular link between BAC exposure and the abnormal epidermal differentiation associated with GP. However, these transcriptional changes should not be interpreted as direct reproduction of the complete histopathological phenotype of parakeratosis in the two-dimensional HaCaT culture system.

## 3. Discussion

In this study, we provide transcriptomic and tissue-level evidence that GP is associated with inflammatory remodeling of the epidermal keratinocyte compartment. By integrating single-cell RNA sequencing, keratinocyte subclustering, pathway enrichment analysis, pseudotime trajectory inference, cell–cell communication analysis, immunofluorescence validation, and an in vitro BAC-exposed keratinocyte model, we identified an inflammatory keratinocyte state enriched in GP lesions and linked this phenotype to aberrant epidermal differentiation. These findings suggest that GP should not be regarded simply as a static disorder of keratinization, but rather as an exposure-associated epidermal differentiation disorder accompanied by inflammatory keratinocyte activation.

The central finding of this study is that inflammatory keratinocytes represent a disease-associated cellular state in GP. Keratinocyte subclustering revealed a shift from homeostatic differentiated keratinocyte populations toward an inflammatory keratinocyte state. These cells expressed genes associated with epidermal activation and innate immune responses. Furthermore, the simultaneous enrichment of inflammatory and differentiation-related pathways is important, as it indicates that inflammatory activation and abnormal terminal differentiation coexist within the same keratinocyte compartment and may represent coordinated features of GP epidermis.

*S100A8* and *S100A9* emerged as representative markers of inflammatory keratinocytes in GP lesions. S100A8 and S100A9 are calcium-binding proteins that function as damage-associated molecular pattern molecules or alarmins in inflamed epithelia [17]. In the skin, they are minimally expressed in normal epidermis but are strongly induced upon keratinocyte activation and epidermal inflammation [18]. Their epidermal upregulation has been reported in inflammatory skin diseases, including psoriasis and atopic dermatitis [19,20,21,22], where they are associated with keratinocyte activation, innate immune amplification, and cytokine-driven inflammation. Therefore, S100A8/S100A9 should not be interpreted as disease-specific biomarkers of GP. In the present study, inflammatory keratinocytes constituted the largest fraction (48.7%) of *S100A8/S100A9* double-positive keratinocytes. Immunofluorescence staining further confirmed increased epidermal expression of both proteins in GP lesions. These findings indicate that *S100A8/S100A9* mark an inflammatory keratinocyte state in GP and support the concept that GP involves inflammatory epidermal remodeling.

Pseudotime analysis further supported an association between inflammatory activation and differentiation-associated keratinocyte states in GP. In healthy skin, basal, spinous, and granular keratinocytes occupied different regions of an interconnected inferred trajectory, along which *S100A8* and *S100A9* expression remained low overall. In GP lesions, inflammatory keratinocytes were concentrated along a trajectory segment spanning regions occupied by spinous and granular keratinocyte states and showed more widespread *S100A8* and *S100A9* expression. These observations are consistent with inflammation-associated remodeling of the keratinocyte differentiation landscape in GP.

Cell–cell communication analysis indicated remodeling of keratinocyte-centered signaling networks in GP. Selected keratinocyte-related ligand–receptor interactions were associated with keratinocyte activation, desmosomal adhesion, epidermal differentiation, and epithelial–immune signaling, suggesting that GP keratinocytes may be involved in the remodeling of epidermal cell–cell adhesion and local epithelial–immune communication.

Previous clinical studies have repeatedly linked GP to exposure to BAC-containing products, but the molecular basis of this association has remained unclear. Our in vitro experiments showed that BAC can act as an upstream stimulus, reducing HaCaT cell viability in a dose-dependent manner and inducing transcriptional programs related to IL-1-associated inflammation, keratinocyte activation, cellular stress, and damage adaptation. GSEA further showed that BAC exposure induced concentration-dependent alterations in keratinocyte differentiation- and keratinization-associated transcriptional programs. This finding is consistent with the established role of keratinocytes as active initiators of cutaneous inflammation. Epidermal injury and barrier disruption can promote keratinocyte IL-1α release, which is sufficient to initiate downstream inflammatory responses in the skin [23,24]. In addition, IL-1 family cytokines are recognized as key mediators of skin inflammation, and keratinocytes can both produce and respond to IL-1-related signals. Thus, BAC-induced IL-1-associated transcriptional activation provides a plausible link between external chemical exposure, keratinocyte stress, and inflammatory epidermal remodeling in GP.

A limitation of this study is the relatively small immunofluorescence validation cohort, which included four patients with GP and three healthy controls. The rarity and potential under-recognition of GP may make the recruitment of larger, well-characterized cohorts challenging. Nevertheless, this sample size may not fully capture the inter-individual and clinicopathological heterogeneity of GP, and therefore limits the generalizability of the tissue-level findings. Larger independent, preferably multicenter, cohorts will be needed to further validate these observations. A further consideration is that parakeratosis is an architecturally defined histopathological phenotype and therefore cannot be fully reproduced in a conventional two-dimensional HaCaT culture system. Accordingly, the HaCaT model in this study was used to characterize BAC-induced molecular changes associated with inflammation, cellular stress, keratinocyte differentiation, and keratinization rather than to reproduce the complete histopathological phenotype of parakeratosis.

In summary, our findings identify inflammatory keratinocyte remodeling as a previously underrecognized feature associated with GP. Rather than being a purely morphological abnormality of cornification, GP may be better understood as an exposure-associated inflammatory epidermal differentiation disorder.

## 4. Materials and Methods

### 4.1. Patient and Sample Information

For the scRNA-Seq discovery cohort, we included three patients with a clinically and histologically confirmed diagnosis of GP and three age- and sex-matched healthy controls. To independently validate the key findings from our scRNA-Seq analysis, we constituted a validation cohort comprising four GP patients and three age- and sex-matched healthy controls. The diagnosis was independently confirmed by two experienced dermatologists. Skin specimens from GP lesions and corresponding sites in healthy controls were used for subsequent analyses.

### 4.2. Analysis of scRNA-Seq Data

Skin biopsies were preserved in tissue preservation solution and transported on ice for processing. Tissues were washed with Hank’s balanced salt solution (HBSS) and digested in tissue dissociation solution at 37 °C for 15 min under gentle agitation. Single-cell libraries were prepared with the Chromium Single Cell 3′ Library Kit (10x Genomics, Pleasanton, CA, USA) according to the manufacturer’s protocol. Briefly, single-cell suspensions were partitioned into droplets using the Chromium Controller, followed by cell lysis, barcoding, and reverse transcription. The constructed libraries were sequenced on an Illumina (San Diego, CA, USA) NovaSeq 6000 system with 150 bp paired-end reads. Raw sequencing data were processed using Cell Ranger (v.3.0.2) with alignment to the GRCh38 reference genome. Subsequent analysis was conducted with Seurat (v.3.0.1), including data normalization, clustering, and the identification of differentially expressed genes. Cell–cell communication was analyzed using the CellChat R package (version 2.1.2) [25]. Normalized single-cell gene expression data and annotated cell identities were used to infer ligand–receptor interactions based on CellChatDB.human. Communication networks were compared between GP and healthy control skin, with particular attention to interactions among keratinocytes, T/NK cells, B/plasma cells, and myeloid cells. Circle plots summarized the numbers of predicted intercellular interactions, while bubble plots displayed the communication probabilities and statistical significance of selected ligand–receptor interactions.

### 4.3. Skin Immunofluorescence Staining and Image Acquisition

Immunofluorescence staining was performed on formalin-fixed, paraffin-embedded (FFPE) human skin tissue sections. The sections were first deparaffinized in xylene and rehydrated through a graded ethanol series. Following antigen retrieval with heat, slides were cooled and treated with a protein blocking solution. Sections were then incubated with primary antibodies against S100A8 (rabbit monoclonal, Cell Signaling Technology, Danvers, MA, USA) and S100A9 (mouse monoclonal, Proteintech, Rosemont, IL, USA). After washing, the sections were incubated with a combination of fluorescent secondary antibodies: Alexa Fluor 594-conjugated antibody (red) for S100A8 detection and Alexa Fluor 488-conjugated antibody (green) for S100A9 detection. Cell nuclei were stained with 4′,6-diamidino-2-phenylindole (DAPI). The slides were then mounted with an anti-fade medium. All incubation steps were carried out at room temperature unless otherwise noted. Fluorescence images were captured from multiple random fields per section at high magnification using a microscope. All images were analyzed in a blinded manner by at least two independent, experienced investigators.

### 4.4. Cell Culture

HaCaT cells obtained from Biovector Science Laboratory (Beijing, China) were cultured in Dulbecco’s modified Eagle medium (DMEM; Cytiva, Marlborough, MA, USA) supplemented with 10% fetal bovine serum (FBS; Gibco, Grand Island, NY, USA) and 1% penicillin–streptomycin (Yeasen Biotechnology, Shanghai, China) under standard culture conditions.

### 4.5. Cell Viability Assay and Bulk RNA Sequencing

To evaluate cell viability, HaCaT cells were treated with different concentrations of BAC (Sigma-Aldrich, St. Louis, MO, USA). Cell viability was determined using the CCK-8 assay (Beyotime Biotechnology, Shanghai, China) according to the manufacturer’s instructions. Based on the viability curve, low- and high-concentration BAC conditions were selected for bulk RNA sequencing. Total RNA was extracted from HaCaT cells using an RNA extraction kit (EZBioscience, Suzhou, China) according to the manufacturer’s instructions. RNA quality was assessed using an Agilent 2100 Bioanalyzer, and samples with an RNA integrity number (RIN) > 7.0 were selected for library construction. The sequencing libraries were prepared following standard protocols and sequenced on an Illumina NovaSeq 6000 platform. Raw sequencing data were processed using standard RNA sequencing analysis pipelines for gene expression quantification and differential expression analysis. Differentially expressed genes (DEGs) were identified using the criteria of |log2 fold change (FC)| > 1 and false discovery rate (FDR) < 0.05.

### 4.6. Statistical Analysis

Statistical analyses were performed using GraphPad Prism 9.0 and R software (v 4.4.3). Data are presented as mean ± standard deviation (SD). Comparisons between two groups were performed using the unpaired Student’s *t*-test. *p* values < 0.05 were considered statistically significant.

## Data Availability

The datasets generated during the current study are available from the corresponding author upon reasonable request.

## Abbreviations

The following abbreviations are used in this manuscript:

GP	Granular parakeratosis
COVID-19	Coronavirus disease 2019
BAC	Benzalkonium chloride
IL-1	Interleukin-1
H&E	Hematoxylin and eosin
scRNA-Seq	Single-cell RNA sequencing
CON	Healthy control
CCK-8	Cell Counting Kit-8
UMAP	Uniform Manifold Approximation and Projection
GO	Gene Ontology
KEGG	Kyoto Encyclopedia of Genes and Genomes
IL-17	Interleukin-17
DAPI	4′,6-Diamidino-2-phenylindole
MIF	Macrophage migration inhibitory factor
LBAC	Low-concentration benzalkonium chloride
HBAC	High-concentration benzalkonium chloride
GSEA	Gene set enrichment analysis
SD	Standard deviation
HBSS	Hank’s balanced salt solution
FFPE	Formalin-fixed, paraffin-embedded
DMEM	Dulbecco’s modified Eagle medium
FBS	Fetal bovine serum
RIN	RNA integrity number
DEGs	Differentially expressed genes
FC	Fold change
NES	Normalized enrichment score
FDR	False discovery rate
